# Bronchioloalveolar invasion in non-small cell lung cancer is associated with expression of transforming growth factor-β1

**DOI:** 10.1186/1477-7819-11-113

**Published:** 2013-05-25

**Authors:** Kazuhiro Imai, Yoshihiro Minamiya, Akiteru Goto, Hiroshi Nanjo, Hajime Saito, Satoru Motoyama, Yusuke Sato, Satoshi Kudo, Shinogu Takashima, Yasushi Kawaharada, Nobuyasu Kurihara, Kimito Orino, Jun-ichi Ogawa

**Affiliations:** 1 Department of Chest, Breast and Endocrine Surgery, Akita University Graduate School of Medicine, 1-1-1 Hondo, Akita City 010-8543, Japan; 2 Department of Pathology, Akita University Graduate School of Medicine, 1-1-1 Hondo, Akita City 010-8543, Japan

**Keywords:** Adenocarcinoma in situ, Lung cancer, Minimally invasive adenocarcinoma, Transforming growth factor-β1

## Abstract

**Background:**

Adenocarcinoma in situ (AIS) and minimally invasive adenocarcinoma (MIA) with fibrous stromal invasion are newly introduced subtypes of small lung adenocarcinoma. AIS is a small localized adenocarcinoma in which growth is restricted to neoplastic cells along preexisting alveolar structures without fibrous stromal invasion. In MIA, by contrast, tumor cells have infiltrated the myofibroblastic stroma. Transforming growth factor (TGF)-β is known to be produced by progressor tumors, and excessive TGF-β contributes to a pathological excess of tissue fibrosis. TGF-β1 is the most abundant isoform, and its expression is a key event fostering tumor invasion and metastasis. We therefore analyzed the relationship between TGF-β1 expression and clinicopathological microinvasion in patients with small lung adenocarcinoma.

**Methods:**

The study participants were 45 patients who underwent curative surgery for AIS and MIA 3 cm or less in size. Those tumors were assessed based on immunohistochemical staining using anti-TGF-β1 antibody. The TGF-β1 status was assessed immunohistochemically using the Allred 8-unit system.

**Results:**

The rates of TGF-β1 positivity in the AIS and MIA groups were 27.3% and 65.2%, respectively (*P* <0.05). The median of Allred score was 0.5 (range 0–5) in the AIS group and 3.0 (range 0–6) in the MIA group (*P* = 0.0017).

**Conclusions:**

We suggest that TGF-β1 expression is likely to be significantly stronger in patients with MIA than in those with AIS, and the increased expression may be associated with minimal invasion and infiltration of the myofibroblastic stroma.

## Background

Adenocarcinoma is the most common histological type among lung cancers. As with malignancies in other organs such as the breast and cervix, where carcinomas are defined as non-invasive, micro-invasive or invasive, the extent of the invasive component in lung adenocarcinomas is associated with clinical outcomes [1]. To address advances in oncology, molecular biology, pathology, radiology, and surgery, an international multidisciplinary classification was sponsored by the International Association for the Study of Lung Cancer (IASLC), the American Thoracic Society, and the European Respiratory Society [2]. Adenocarcinoma in situ (AIS) and minimally invasive adenocarcinoma (MIA) with fibrous stromal invasion are newly introduced subtypes, while the older term of bronchioloalveolar carcinoma does no longer exist according to new IASLC classification of lung adenocarcinoma. AIS is a localized small (≤3 cm) adenocarcinoma, the growth of which is restricted to neoplastic cells along preexisting alveolar structures without stromal, vascular or pleural invasion. Papillary or micropapillary patterns and intra-alveolar tumor cells are absent. MIA is a small, solitary adenocarcinoma growing in a predominantly lepidic pattern and showing ≤5 mm invasion in its greatest dimension at any one focus. If these tumors are completely resected, there will be 100% or near 100% disease-specific survival.

Transforming growth factor (TGF)-β reportedly promotes cancer metastasis by affecting the tumor microenvironment in a manner that facilitates tumor cell invasion [3,4]. Several tumors, including those arising in the lung [5-7], express high levels of TGF-β, which correlate with tumor progression and clinical prognosis. Humans express three highly homologous isoforms of TGF-β (TGF-βs 1, 2, and 3), which share 70% sequence identity in their biologically active C-terminal regions, and all of which bind to the same receptor complex and activate the same downstream signaling pathways. TGF-β1 is the most abundant and most extensively studied isoform [8].

We previously showed that tumor-derived TGF-β1 causes a reduction in the number of dendritic cells within the sentinel lymph node in lung cancer [9]. Our findings also suggest that TGF-β1 29T>C genetic polymorphism is associated with lymph node metastasis in patients with adenocarcinoma of the lung [10]. In addition, we showed that overexpression of TGF-β1 by tumor cells promotes metastasis into tumor-draining lymph nodes in mice, most likely by inhibiting dendritic cell migration from tumors towards tumor-draining nodes [11]. Collectively, these results suggest that TGF-β1 is a key mediator fostering tumor invasion and metastasis. We therefore analyzed the relationship between TGF-β1 expression and clinicopathological microinvasion in patients with small lung adenocarcinoma.

## Methods

### Patients

In total, 453 patients who had undergone major pulmonary resections for non-small cell lung cancer (NSCLC) were enrolled in the study. Among them, 45 patients (10.1%) with NSCLC had AIS and MIA subtypes of small lung adenocarcinoma 3 cm or less in size. All had undergone surgery in the Department of Chest, Breast and Endocrine Surgery, University Hospital of Akita University Graduate School of Medicine, between January 2004 and December 2011. This study was approved by our institutional review boards, and written informed consent was obtained from all patients. The patients’ characteristics are listed in Table 1. The 7th edition of the TNM staging system was used for evaluation [12].

**Table 1:** Clinical details of all patients who underwent pulmonary resection for small lung adenocarcinoma

	**AIS**	**MIA**	***P *value**
n	22	23	
Age (year)	63±11.9	67±9.3	0.232
Gender (M/F)	8/14	8/15	0.675
Tumor size	11.1±3.7	15.0±5.6	0.022*
Nodal involvement			0.162
N0	22	22	
N1	0	1	
N2	0	0	
Stage			0.243
IA	22	22	
IB	0	0	
IIA	0	1	

*AIS* Adenocarcinoma in situ, *MIA* Minimally invasive adenocarcinoma, *P <0.05.

### Pathology

**Figure 1:**
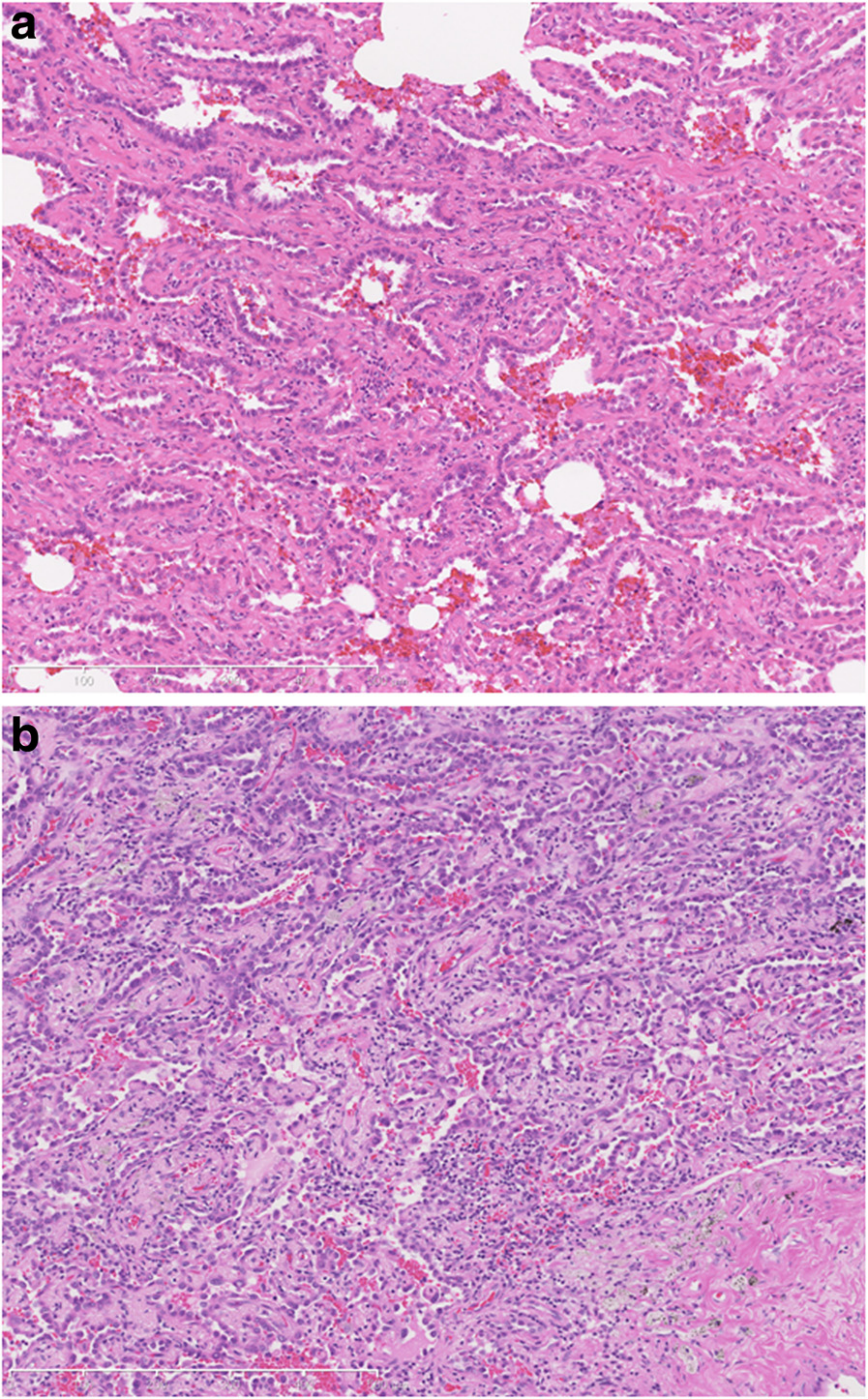
**International multidisciplinary classification of lung adenocarcinoma.** (**a**) Adenocarcinoma in situ. Histologic specimen showing a tumor with atypical pneumocytes proliferating along slightly thickened but preserved alveolar walls; (**b**) Minimally invasive adenocarcinoma. Histologic specimen of a tumor exhibiting a bronchioloalveolar growth pattern with minimal invasion. The tumor is invading in the fibrous stroma.

Surgically resected specimens were fixed in 10% formalin and routinely processed for paraffin embedding. Histological sections were cut into 4-mm slices, which were then stained with hematoxylin and eosin (HE) and elastica Masson using standard methods, and were reviewed by two pathologists (A.G. and H.N.). Experienced pathologists diagnosed the subtypes of the primary tumors according to IASLC/American Thoracic Society/European Respiratory Society International Multidisciplinary Classification of adenocarcinoma [2]. Diagnosis of AIS or MIA was based on the HE staining (Figure 1).

### Immunohistochemistry of TGF-β1 in small lung adenocarcinoma

**Figure 2:**
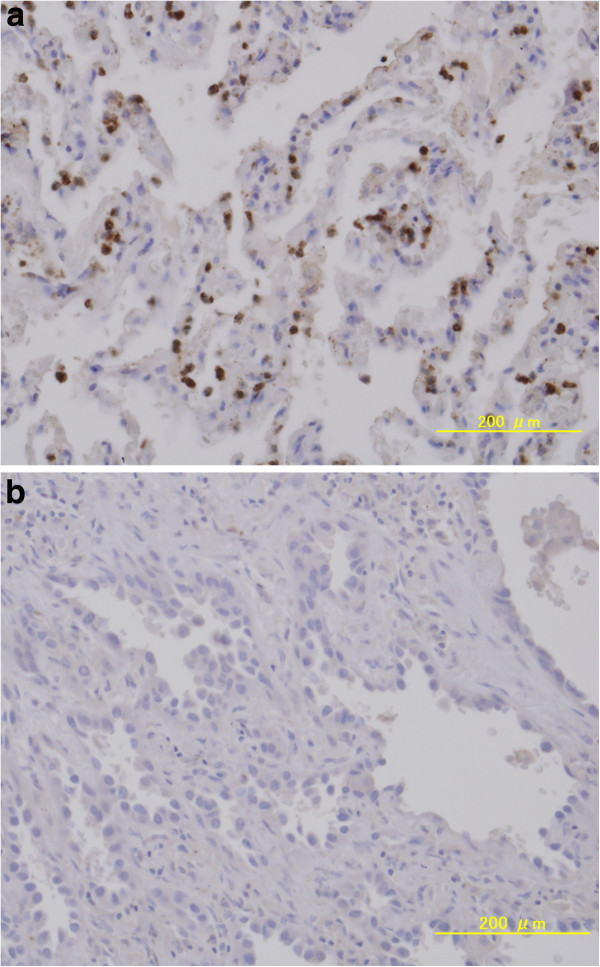
**Immunostaining adenocarcinoma in situ and minimally invasive adenocarcinoma using an anti-TGF-β1 antibody.** (**a**) TGF-β1-positive MIA sample. TGF-β1-positive cells are stained brown. TGF-β1-positive cells are predominantly observed in the tumor itself. This sample has an Allred score of 6/8; (**b**) TGF-β1-negative AIS sample. This sample has an Allred score of 0/8. TGF: Transforming growth factor; AIS: Adenocarcinoma in situ; MIA: Minimally invasive adenocarcinoma.

After reviewing HE-stained sections of the tumor specimens, we selected blocks from the central regions of the tumors for further study. The paraffin-embedded tumor tissues were cut into 4-μm-thick sections and deparaffinized. Small lung adenocarcinomas were then assessed based on standard immunohistochemical (IHC) staining using goat polyclonal anti-TGF-β1 (1:50 dilution; Santa Cruz Biotechnology, Inc., Santa Cruz, CA, USA), anti-goat horseradish peroxidase (1:100 dilution), and diaminobenzidene stain (Figure 2). The TGF-β1 staining was scored using the Allred 8-unit system with the combination of a proportion score from 0 to 5 and an intensity score from 0 to 3. The proportion score included the fraction of positively stained tumor cells and was as follows: 0 = none, 1 ≤1/100; 2 = 1/100 to 1/10; 3 = 1/10 to 1/3; 4 = 1/3 to 2/3; 5 ≥2/3. The staining intensity score was as follows: 0 = none; 1 = weak; 2 = intermediate; 3 = strong [13,14].

### Immunohistochemical detection of micrometastasis and isolated tumor cells in dissected lymph nodes

Isolated tumor cells and lymph node micrometastasis were assessed in all patients based on HE staining and IHC using AE1/AE3 antibodies. One section of the maximum cut surface of each lymph node was immunohistochemically labeled with AE1/AE3 monoclonal mouse anti-human cytokeratin clones using an EnVision system (DAKO Corporation, Carpinteria, CA, USA), which was used to detect the presence of micrometastases and isolated tumor cells. A result was considered positive if positive cell clusters or individual cells with the appropriate tumor cell morphology were recognized. As proposed by the 7^th^ edition of the TNM staging system [12], isolated tumor cells were not considered as positive, but were defined as pN0(i+) in this study.

### Statistics

Group data were expressed as means ± SD. Categorical data were compared using the χ^2^ test. The significance of individual differences was evaluated using the Wilcoxon test. Values of *P* <0.05 were considered to be significant. JMP IN 8.0.2 software (SAS Institute, Cary, NC, USA) was used for all statistical evaluations.

## Results

**Figure 3:**
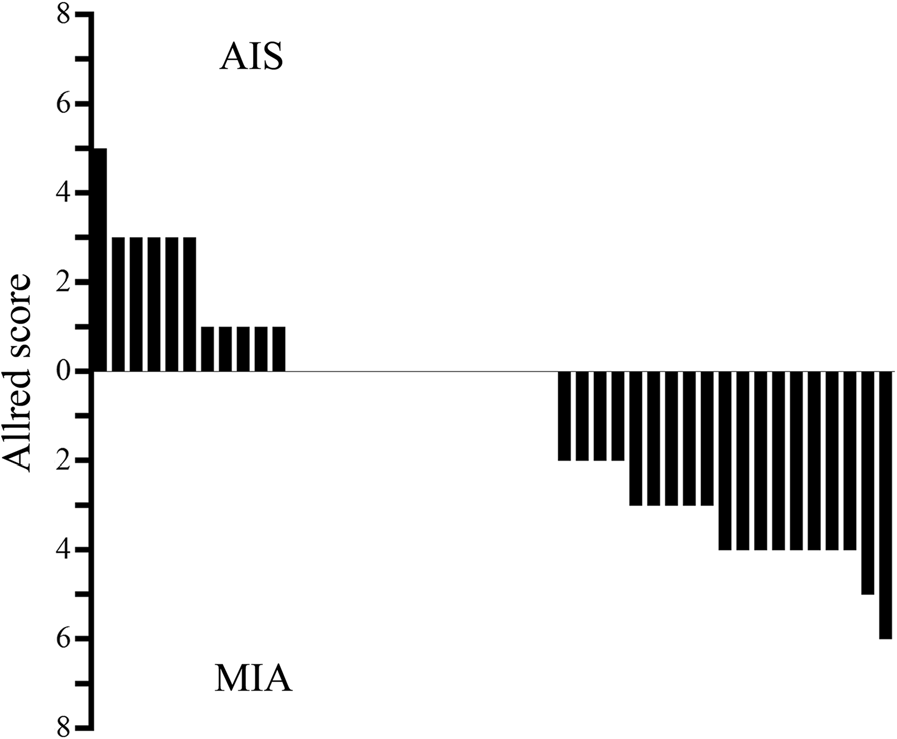
**Allred score system devised to score results from immunohistochemical tests of TGF-β1.** Differences in TGF-β1 immunostaining between AIS and MIA scored using the Allred method; *P <0.05.

There were no differences between the AIS and MIA groups with respect to age, gender, nodal involvement or pathological stage; however, tumor size was greater in the MIA group than the AIS group (Table 1). Table 2 shows the incidence of TGF-β1 expression detected upon immunohistochemical examination in the AIS and MIA groups. Figure 3 shows the differences in TGF-β1 immunostaining between AIS and MIA scored using the Allred method. The rate of TGF-β1 positivity was significantly (*P* <0.05) higher in the MIA group (65.2%; 15/23) than the AIS group (27.3%; 6/22). Similarly, the median Allred score for TGF-β1 expression was significantly higher (*P* = 0.0017) in the MIA group than the AIS group (3.0 (range 0–6) *vs.* 0.5 (range 0–5)).

**Table 2:** Incidence of TGF-β1 expression in the AIS and MIA groups

	**TGF-β1 expression positive stain (%)**
AIS (n=22)	27.3
MIA (n=23)	65.2*

*AIS* Adenocarcinoma in situ, *MIA* Minimally invasive adenocarcinoma, *P <0.05.

**Figure 4:**
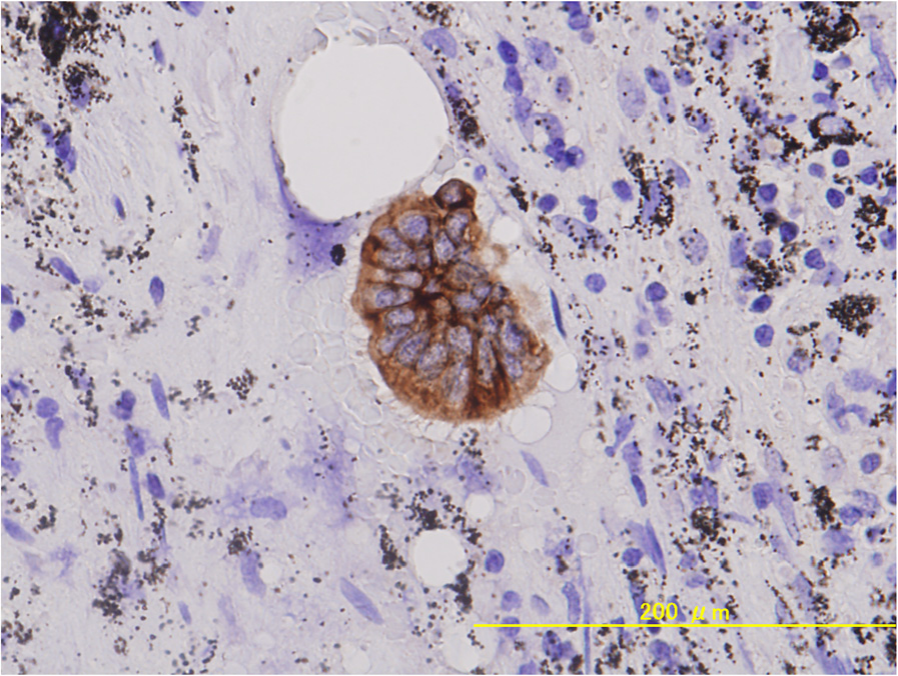
**Isolated tumor cells in a dissected lymph node.** Isolated tumor cells were detected by immunostaining using anti-cytokeratin antibody in a patient diagnosed with MIA. The patient’s pathological stage was pT1aN(i+)M0, Stage IA.

One instance of lymph node metastasis was detected in the MIA group using HE and IHC staining with AE1/AE3 antibodies. In addition, isolated tumor cells were found in a second patient diagnosed with MIA (Figure 4). This patient was staged as pT1aN0(i+)M0. The overall survival rates differed somewhat between AIS and MIA patients (100% *vs.* 89.5%), but that difference did not reach statistical significance (*P* = 0.093).

## Discussion

The results of the present study suggest that patients with MIA show greater expression of TGF-β1 than patients with AIS, as indicated by the significantly greater TGF-β1 positivity rate and Allred score. In addition, HE and IHC staining revealed that lymph node metastasis and isolated tumor cells were only present in the MIA group. Thus, TGF-β1 expression by adenocarcinomas appears to be an important factor associated with clinicopathological microinvasion in patients with small lung adenocarcinoma.

TGF-β increases the invasiveness of cancer cells by increasing their proteolytic activity and promoting their binding to cell-adhesion molecules [15]. In an earlier study, we demonstrated that tumor-derived TGF-β1 induces dendritic cell apoptosis within lymph nodes in non-small cell lung cancer [9], and that overexpression of TGF-β1 by tumor cells promotes lymph node metastasis in mice [11]. Consistent with those findings, the effects of TGF-β1 on angiogenesis, stroma formation and immune function appear to further support tumor progression and invasion [16,17]. AIS is a small localized adenocarcinoma in which growth is restricted to neoplastic cells along preexisting alveolar structures without fibrous stromal invasion. In MIA, by contrast, tumor cells have infiltrated the myofibroblastic stroma. In many diseases, excessive TGF-β contributes to a pathological excess of tissue fibrosis that compromises normal organ function, a topic that has been the subject of several reviews [18-20]. However, we provide no direct evidence of the mechanism by which TGF-β1 increases tissue fibrosis in MIAs.

The Noguchi classification [21] is predictive of outcome in patients with small adenocarcinomas, with type D, E, and F tumors showing a worse outcome than the other tumor types. However, the majority of small adenocarcinomas are classified as type C, so that subclassification of type C tumors does seem necessary. Tumors that meet the criteria for AIS were formerly classified as bronchioloalveolar carcinoma based on the strict definition of type A and type B adenocarcinomas from the 1995 Noguchi classification [21]. MIAs are tumors showing lepidic growth and a small (≤5 mm) area of invasion. The MIA invades areas of stromal fibrosis in an acinar pattern. Nearly all type C adenocarcinomas can be classified as MIAs. The outcome of patients with adenocarcinomas with diameters of 2 cm or less can be predicted using the following scoring system based on three histological criteria: 1) the tumor is lymphovascular invasion-positive; 2) the non-bronchioloalveolar carcinoma component is >10 mm in diameter; and 3) the percentage of the solid, cribriform, and/or papillary components is ≥30% of the entire tumor volume [22]. Patients whose MIAs have a non-bronchioloalveolar carcinoma component ≤5 mm show 100% survival [22,23]. Consistent with that finding, no difference in survival was observed between AIS and MIA in the present study. However, nodal involvement was observed in 2 of 23 patients with MIA. We therefore suggest that it would be problematic to conduct limited surgery for MIA patients with micrometastasis or isolated tumor cells, though some MIAs may be candidates for limited surgery.

## Conclusions

In conclusion, our findings suggest that patients with MIAs have both a significantly greater rate of TGF-β1 positivity and higher TGF-β1 Allred scores than patients with AIS. However, because we do not provide direct evidence of the mechanism by which TGF-β1 increases tissue fibrosis in MIAs, and we examined only a limited number of patients, further studies will be needed to more precisely define the role played by tumor-derived TGF-β1 in determining the invasiveness of adenocarcinoma of the lung.

## Abbreviations

AIS: Adenocarcinoma in situ; HE: hematoxylin and eosin; IHC: immunohistochemical; MIA: Minimally invasive adenocarcinoma; NSCLC: Non-small cell lung cancer; TGF: Transforming growth factor.

## Competing interests

The authors declare that they have no competing interests.

## Authors' contributions

KI performed immunohistochemical staining, analyzed data and wrote the paper. YM designed the research. AG and HN evaluated histological staining and diagnosed lymph node metastasis. HS, SM, YS, SK, ST, YK, and NK performed and contributed to data analysis. KO contributed to histological diagnosis. JO contributed to clinical design and data analysis. All authors read and approved the final manuscript.
